# Comparative outcomes across rituximab induction doses in pemphigus: A real-world single-center study

**DOI:** 10.1016/j.jdin.2025.11.007

**Published:** 2025-11-19

**Authors:** Yujia Wang, Liangchun Wang, Yanyun Jiang

**Affiliations:** Department of Dermatology, Sun Yat-sen Memorial Hospital, Sun Yat-sen University, Guangzhou, China

**Keywords:** autoimmune blistering disease, corticosteroids, dosing strategy, epidemiology, pemphigus, rituximab, therapy

*To the Editor:* Pemphigus vulgaris is an autoimmune blistering disorder that can cause erosions of the skin and mucous membranes. Corticosteroids (CS) remain the primary treatment, but long-term use may lead to significant adverse effects. Immunosuppressants can reduce CS dependence but carry infection risks and limited efficacy. Rituximab (RTX), an anti-CD20 monoclonal antibody, has emerged as an effective option, improving remission rates and reducing CS dependence.^1^ However, the optimal dosing strategy remains uncertain. While high-dose RTX is effective, it is costly and may increase infection risk. Low-dose and ultralow-dose regimens are gaining attention but their durability remains under evaluation.^2^^,^^3^ In China, dosing decisions are often influenced by economic factors and physician discretion. Our study conducted a retrospective summary of the clinical data of pemphigus patients treated with different doses of RTX in our center.

We retrospectively analyzed 53 pemphigus patients treated with RTX at Sun Yat-sen Memorial Hospital between November 2021 and September 2024. Patients were stratified by total induction dose in the first month: high-dose RTX (≥1000 mg), low-dose RTX (200-1000 mg), and ultralow-dose RTX (≤200 mg). All patients were followed for at least 12 months.

The detailed information of these 53 patients was summarized in Supplementary Material, available via Mendeley at https://data.mendeley.com/datasets/hwh3cn3wsw/1 and the baseline characteristics in the groups described in Table I. Baseline characteristics were similar among groups, except for prior treatment regimens (*P* = .014). Complete remission (CR),^4^ which served as the primary endpoint, and relapse were evaluated at 6 and 12 months. In both unadjusted and adjusted analyses controlling for prior treatment, no significant differences in CR or relapse rates were observed among the groups (Table II). Kaplan-Meier analysis revealed no significant differences in time to CR or relapse rates during early and late follow-up (log-rank *P* = .931 and *P* = .806, respectively, Supplementary Material, available via Mendeley at https://data.mendeley.com/datasets/hwh3cn3wsw/1). Although cumulative CS usage was numerically lower in the low-dose RTX group, the difference was not significant (median [interquartile range], mg: 3194 (2377) vs 2715 (2262) vs 3233 (2965); *P* = .482).

**Table I:** Baseline characteristics among 3 groups

	RTX			*P* value
	ULRTX, *n* = 15	LRTX, *n* = 18	HRTX, *n* = 20	
Age, mean ± SD, y	51 ± 15	54 ± 12	46 ± 16	.399
Sex, *n* (%)				.819
Male	7 (47)	9 (50)	8 (40)	
Female	8 (53)	9 (50)	12 (60)	
Diagnose, *n* (%)				.396
PV	13 (87)	17 (94)	16 (80)	
PF	2 (13)	1 (6)	4 (20)	
PDAI, median (IQR)	21 (13-30)	23 (15-33)	37 (20-42)	.092
Disease severity, *n* (%)				.273
Mild	1 (7)	2 (12)	2 (11)	
Moderate	9 (60)	7 (41)	4 (22)	
Severe	5 (33)	8 (47)	12 (68)	
Disease duration, median (IQR), mo	34 (7-48)	28 (10-48)	12 (3-32)	.233
Previous treatment, *n* (%)				.014
CS only	13 (87)	8 (44)	16 (80)	
CS with ISD	2 (13)	10 (56)	4 (20)	
Prednisolone dose∗, mean ± SD, mg/d	35 ± 19	46 ± 29	53 ± 32	.192
Anti-Dsg level∗, U/mL				
Dsg1, median (IQR)	200 (171-200)	188 (55-200)	200 (163-200)	.700
Dsg3, median (IQR)	157 (4-200)	200 (73-200)	200 (74-200)	.443
B cells∗, mean ± SD, %	13 ± 8	16 ± 8	16 ± 6	.496

*Anti-Dsg*, Antidesmoglein antibodies; *CS*, corticosteroids; *HRTX*, high-dose rituximab; *IQR*, interquartile range; *ISD*, immunosuppressive drugs; *LRTX*, low-dose rituximab; *PDAI*, pemphigus disease area index; *PF*, pemphigus foliaceus; *PV*, pemphigus vulgaris; *RTX*, rituximab; *SD*, standard deviation; *ULRTX*, ultra-low dose rituximab*n*.

∗ All variables represent previous values.

**Table II:** Clinical outcomes of 6 months and 12 months among 3 groups

Outcomes	Groups	Unadjusted OR (95% CI)	*P* value	Adjusted OR∗ (95% CI)	*P* value
6 mo					
CR, *n* (%)	ULRTX	Ref		Ref	
	LRTX	1.11 (0.26-4.72)	.886	1.01 (0.21-4.83)	.988
	HRTX	1.44 (0.35-5.95)	.611	1.42 (0.34-5.88)	.627
Relapse, *n* (%)	ULRTX	Ref		Ref	
	LRTX	0.81 (0.15-4.40)	.810	1.00 (0.16-6.43)	.999
	HRTX	1.42 (0.24-8.26)	.699	1.47 (0.25-8.62)	.673
12 mo					
CR, *n* (%)	ULRTX	Ref		Ref	
	LRTX	1.29 (0.32-5.17)	.723	1.34 (0.29-6.18)	.709
	HRTX	0.53 (0.13-2.14)	.371	0.53 (0.13-2.15)	.373
Relapse, *n* (%)	ULRTX	Ref		Ref	
	LRTX	0.67 (0.15-3.04)	.600	0.89 (0.17-4.76)	.895
	HRTX	1.02 (0.22-4.72)	.982	1.04 (0.22-4.85)	.964

*CI*, Confidence interval; *CR*, complete remission; *HRTX*, high-dose rituximab; *LRTX*, low-dose rituximab; *OR*, odds ratio; *Ref*, reference; *RTX*, rituximab.

∗ Adjusted OR (95% CI) calculated after adjustment for previous treatment.

All regimens achieved near-complete peripheral B-cell depletion at 1 month, accompanied by a sustained overall decline in antidesmoglein antibody levels, with no statistically significant differences between groups (69% vs 70% vs 77%, *P* = .874). Adverse events (AEs) were mostly mild, with no statistically significant differences in incidence among the groups (20% vs 33% vs 30%, *P* = .586). Edema was most common, and 1 severe pulmonary infection occurred in the high-dose RTX group. Other AEs included hyperglycemia, hypertension, arthralgia, femoral head necrosis, folliculitis, and pruritus.

Our findings indicate that RTX is effective and generally well tolerated in pemphigus across different dosing strategies, in line with results from recent prospective studies.^3^ AE rates appeared lower in the ultralow-dose RTX group without statistical significance, suggesting a possible trend. Limitations of this study include the small sample size, heterogeneous follow-up durations, and the absence of standardized long-term immunologic monitoring. Larger prospective multicenter studies are warranted to validate whether low-dose RTX protocols can maintain durable disease control with improved safety and cost-effectiveness.

## Conflicts of interest

None disclosed.
